# Identification of Aldehyde Dehydrogenase Gene Family in *Glycyrrhiza uralensis* and Analysis of Expression Pattern Under Drought Stress

**DOI:** 10.3390/ijms26052333

**Published:** 2025-03-05

**Authors:** Mengyuan He, Xu Ouyang, Linyuan Cheng, Yuetao Li, Nana Shi, Hongxia Ma, Yu Sun, Hua Yao, Haitao Shen

**Affiliations:** 1 Key Laboratory of Xinjiang Phytomedicine Resource and Utilization of Ministry of Education, College of Life Sciences, Shihezi University, Shihezi 832003, China; hemengyuan1999@163.com (M.H.); 17734921946@163.com (X.O.); c19109354382@163.com (L.C.); lyt18715962753@163.com (Y.L.); 13209988724@163.com (N.S.); 15026231269@163.com (H.M.); sundayu150214@163.com (Y.S.); 2 Key Laboratory of Oasis Town and Mountain-Basin System Ecology of Xinjiang Production and Construction Corps, Shihezi University, Shihezi 832003, China

**Keywords:** *ALDH*, drought stress, *Glycyrrhiza uralensis*, gene family

## Abstract

*Aldehyde dehydrogenases* (*ALDHs*) are a gene family that relies on NAD +/NADP + proteins to oxidize toxic aldehydes to non-toxic carboxylic acids, and they play a crucial role in the growth and development of plants, as well as in their ability to withstand stress. This study identified 26 *ALDH* genes from six *Glycyrrhiza uralensis* gene families distributed on six chromosomes. By analyzing the phylogeny, gene structure, conserved motifs, cis-regulatory elements, collinearity of homologs, evolutionary patterns, differentiation patterns, and expression variations under drought stress, we found that the *ALDH* gene is involved in phytohormones and exhibits responsiveness to various environmental stressors by modulating multiple cis-regulatory elements. In addition, *GuALDH3H1*, *GuALDH6B1*, *GuALDH12A2*, and *GuALDH12A1* have been identified as playing a crucial role in the response to drought stress. By analyzing the expression patterns of different tissues under drought stress, we discovered that *GuALDH3I2* and *GuALDH2B2* exhibited the most pronounced impact in relation to the drought stress response, which indicates that they play a positive role in the response to abiotic stress. These findings provide a comprehensive theoretical basis for the *ALDH* gene family in *Glycyrrhiza uralensis* and enhance our understanding of the molecular mechanisms underlying *ALDH* genes in licorice growth, development, and adaptation to drought stress.

## 1. Introduction

*Glycyrrhiza uralensis* is commonly found in arid and semi-arid areas in Northwest China. It is a bulk herb used for both medicine and feeding, and it is also a pioneer plant for windbreak and sand fixation. The molecular mechanism of plant response to abiotic stress involves multiple aspects, including sensing, signal transduction, transcription, translation, and modification [1]. Plants can maintain normal growth by regulating their development and structure, synthesizing secondary metabolites to cope with stress, and scavenging free radicals. In this process, the main strategies plants use to cope with stress are removing excessive aldehyde molecules and reducing the production of reactive oxygen species with the aim of protecting the normal functions of cell membranes and proteins [2,3,4].

Aldehydes, as an indispensable intermediate product in the catabolism and biosynthesis pathways of organisms, increase significantly in response to abiotic stress. However, once the accumulation of aldehydes is beyond limits, it can cause a potentially toxic effect on the normal growth of plants [5]. In contrast, the *Aldehyde dehydrogenase* (*ALDH*) superfamily possesses the capability to catalyze the conversion of aldehyde compounds into their respective carboxylic acids. Under certain conditions, as an “aldehyde scavenger”, the *ALDH* enzyme acts as a metabolized active aldehyde of lipid peroxidation-derived aldehydes, which may exhibit toxicity as a result of their high reactivity with nucleophilic substances (e.g., nucleic acids, proteins, and membrane lipids) [6,7].

It has been demonstrated that the *ALDH* gene family is widely present in eukaryotes and prokaryotes, and 24 distinct gene families have been identified for quite some time [8], of which eukaryotes contain 20 *ALDH* gene families [9]. Fourteen *ALDH* families have been mentioned in various species of plants, but the *ALDH23* and *ALDH24* gene families have only been found in *Chlamydomonas reinhardtii*, *Physcomitrella patens*, and *Selaginella moellendorffii* [10]. Additionally, *ALDH* proteins are located in the cytoplasm, mitochondria, plastids (chloroplasts, chromatin, and leuplasta), peroxisomes, and microsomes in plants [11].

*ALDHs* play significant roles in plant growth and development, as well as in the response to phytohormones and environmental stressors. Studies in the model organism *Arabidopsis thaliana* indicated that 14 *ALDH* proteins from nine distinct families have been authenticated [12]. The *AtALDH3H1* and *AtALDH7B4* genes are activated in response to drought, salt stress, and the plant hormone abscisic acid (ABA), contributing to the salt tolerance and drought tolerance observed in *Arabidopsis thaliana* [6,13]. The 53 *ALDH* genes classified into 10 families have been documented in soybeans. Notably, the *ALDH2* and *ALDH3* gene families are crucial for responding to drought stress [14]. Our findings indicate that under drought conditions, the expression of the *ALDH7B1* gene is significantly up-regulated in both the roots and leaves of soybean plants; we also observed a marked increase in the expression levels of several other genes, specifically *ALDH2B1*, *ALDH2B2*, *ALDH3H2*, *ALDH12A2*, and *ALDH18B3*, in leaf tissues. In peanuts, aggregately, 71 members of the *ALDH* family were identified across 10 distinct families exhibiting similar structural models and gene architectures. Under drought stress conditions, specific *AhALDhs* members located in roots—including *AhALD10A1*, *AhALD22A1*, *AhALD12A1*, and *AhALD6B2*—are actively involved in mediating plant responses to adverse environmental conditions [15].

The 20 *ALDH* genes found in rice can be categorized into ten gene families based on their protein sequences. Previous studies have demonstrated that the remodeling of rice genome expression under drought, salt, and ABA stresses significantly reflects organ-specific characteristics when exposed to drought and high salinity conditions [16]. The expression levels of five specific genes, *OsALDH2-4*, *OsALDH3-4*, *OsALDH7*, *OsALDH18-2*, and *OsALDH12*, were found to be significantly up-regulated by more than two-fold in seedlings subjected to drought stress, thereby confirming the organ specificity of gene expression [17]. In another tissue, the *ALDH2B* gene exhibited pronounced expression characteristics. However, when *OsALDH2B* is mutated, this phenomenon leads to the occurrence of premature degradation of pollen mother cells accompanied with their abnormal development [18].

As an important component in regulating the transcriptional expression of biological genes, it activates or inhibits gene expression by interacting with cis-acting elements in the gene promoter region. It participates in the regulation of life activities within biological cells. In previous studies, we found that transcription factors play a significant role in mitigating the adverse effects of drought stress on plants. Among them, *MYB*, as the most widespread family in plants, can resist the impacts of drought and cold stresses to some extent [19]. The transcription factor *bZIP* enhances stability and activity after post-translational modifications, allowing it to respond to stimuli inside and outside plant cells [20]. Recent research has shown that in grapes, *NAC* transcription factors enhance the response of transgenic *Arabidopsis* to drought and salt stress by regulating the synthesis of jasmonic acid [21]. *ALDH* proteins have been extensively studied across various plant species and play significant roles in *Arabidopsis thaliana* (thale cress) [22], *Vitis vinifera* (grapevine) [23], *Sorghum bicolor* (sorghum) [24], *Oryza sativa* (rice) [25], maize [26], *Glycine max* (soybean) [14], cotton [27], apple [28], and potato [29]. These studies indicate that the *ALDH* gene plays a crucial role in plants’ tolerance to abiotic stress. The genome-wide study of the *ALDH* gene family in response to environmental stresses provides a theoretical basis for the precise regulation of *Glycyrrhiza uralensis*’s tolerance against various environmental stresses. Research on how the *ALDH* gene family in *Glycyrrhiza uralensis* responds to environmental stress is still in its early stages. This study utilized wild-type *Glycyrrhiza uralensis* to examine the protein structure and function of the *ALDH* gene family and to analyze aspects such as phylogenetic relationships, gene structure characteristics, homology, and cis-regulatory elements. The expression patterns of these genes in various tissues under drought stress were also investigated. Key stress response genes were identified, and their co-expression regulatory networks were analyzed. This research aims to provide a theoretical foundation for the further exploration of the molecular mechanisms by which *ALDH* contributes to drought stress in *Glycyrrhiza uralensis*.

## 2. Results

### 2.1. The Identification and Analysis of the ALDH Gene in the Whole Genome of Glycyrrhiza uralensis

In the PFAM database, a conserved domain of the *ALDH* protein (PF00171) was utilized in the Hidden Markov Model (HMM) to screen for *ALDH* proteins in the complete genome of *Glycyrrhiza uralensis*. This study identified 26 *ALDH* proteins in *Glycyrrhiza uralensis*, which were classified into six families based on the phylogenetic relationships of their protein sequences with those of *Arabidopsis ALDH*. The fundamental features of these proteins, such as the length of the coding sequence, the length of the amino acids, molecular weight, isoelectric point, and subcellular localization, were examined (Table 1). The amino acid lengths of *GuALDH* varied between 138 and 727 residues, while the molecular weights were observed to range from 15.39 to 79.45 kDa. The theoretical isoelectric points of *GuALDH* proteins spanned from 5.13 to 10.06. The majority of *GuALDH* proteins were found to be localized in the cytoplasm (Table 1). Two of the six *ALDH* families contained more than five genes (*ALDH2*, eight genes; *ALDH3*, nine genes), while the other four families contained no more than five genes (*ALDH6*, two genes; *ALDH10*, two genes; *ALDH11*, three genes; *ALDH12*, two genes).

**Figure 1:**
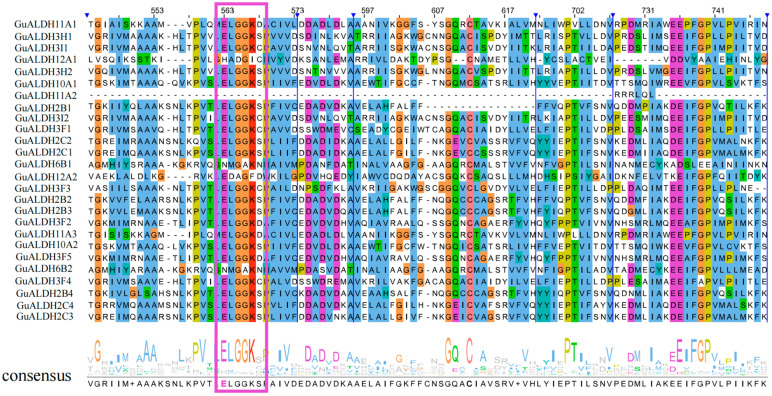
The sequence alignment of the *ALDH* conserved structural domain of the *GuALDH* proteins. The conserved *ALDH* domain (PF00171) of all *GuALDH* proteins was analyzed. The purple frame indicates the conserved glutamic acid active site (PS00687), and the purple label indicates the conserved active site.

The *ALDH* protein superfamily possesses a conserved domain (PF00171), which is characterized by a structural domain that contains distinct sites for catalysis, cofactor binding, and polymerization. These sites have the capacity to function independently or in concert with one another. Then, multiple sequence alignment was performed using Jalview software (Vers. 2.11.2.4) to confirm the glutamate active site in the *GuALDH* protein (PS00687). In *Glycyrrhiza uralensis*, 26 *GuALDH* proteins contain glutamate active sites (Figure 1). Other proteins lack these active sites, probably because they are incomplete sequences. However, searching for functional domains in the NCBI indicated that these genes are still part of the *ALDH* superfamily.

### 2.2. Phylogenetic Analysis of ALDHs

**Figure 2:**
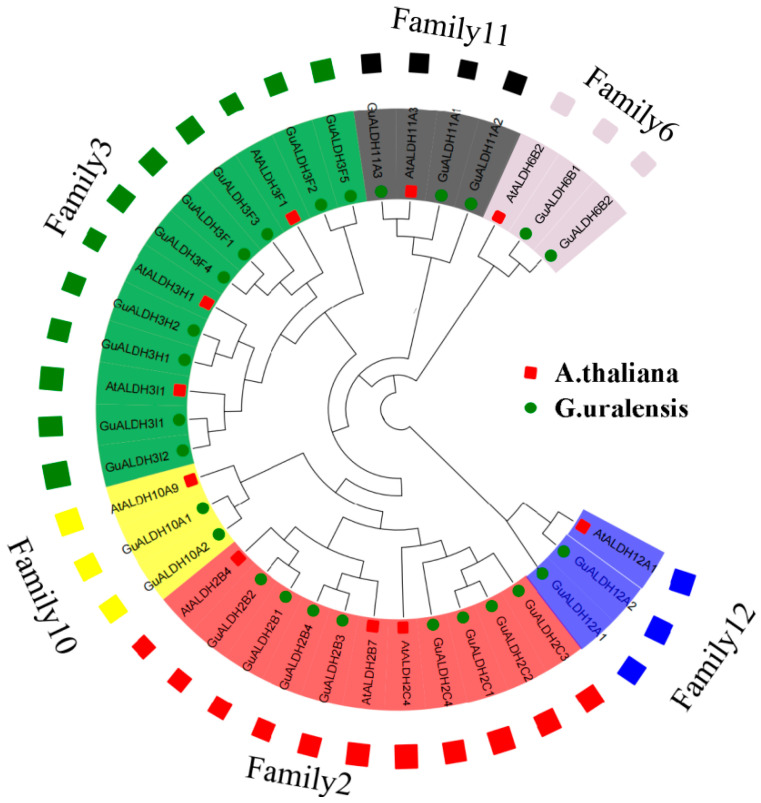
Phylogenetic analysis of *ALDH* members. *ALDH* proteins were aligned using ClustalW, and phylogenetic analysis was performed with MEGA11 based on maximum likelihood. Resulting tree was categorized into six families, with each background color representing different family and specific name of each *ALDH* family labeled accordingly. Green star indicates *Glycyrrhiza uralensis*, and red star indicates *Arabidopsis thaliana*.

To uncover the genetic evolutionary relationship of *ALDH* proteins between *Glycyrrhiza uralensis* and *Arabidopsis thaliana*, the maximum likelihood method was employed to conduct an analysis of the *ALDH* proteins in both species, with 1000 bootstrap replicates being conducted (Figure 2). The names of the *ALDH* proteins in *Glycyrrhiza uralensis* were according to *Arabidopsis*, the phylogenetic tree, and the anterior–posterior relationship of their gene IDs (Figure 2).

The phylogenetic tree revealed that *ALDH2* (11 members) and *ALDH3* (12 members) are the largest clusters, while *ALDH10* (3 members), *ALDH12* (3 members), and *ALDH6* (3 members) are the smallest families. In addition, *Glycyrrhiza uralensis* contains the highest number of members in the third family. We identified two sets of directly related orthologous genes in the *ALDH* family of *Glycyrrhiza uralensis* and the *ALDH* family of *Arabidopsis*: *GuALDH11A3*/*AtALDH11A3* and *GuALDH12A2*/*AtALDH12A1*. These findings suggest that *ALDH* family 3 in *Glycyrrhiza uralensis* may have existed for a considerable duration, and it performs an essential function in the plant’s growth, development, and ability to withstand environmental stresses.

### 2.3. Analysis of Gene Structure and Conserved Protein Moift of GuALDH Subfamily Members in Glycyrrhiza uralensis

**Figure 3:**
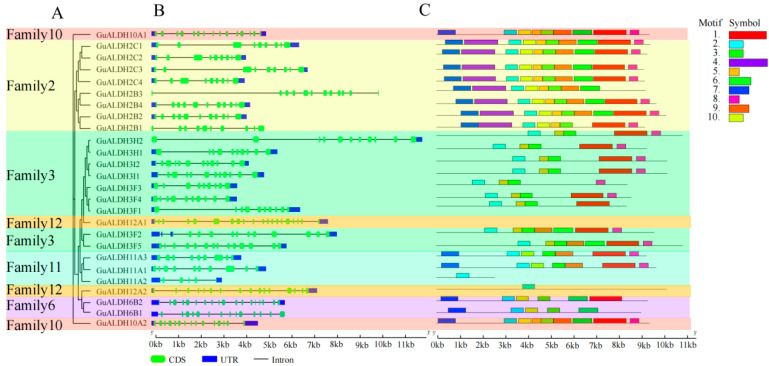
The structure and protein structure of the *ALDH* gene family in *Glycyrrhiza uralensis*. (**A**) The phylogenetic tree was created using MEGA11. (**B**) A structural analysis of the exons/introns of *GuALDH* genes. The green box indicates the exon, and the blue box indicates the 3′ or 5′ UTRs (untranslated regions). (**C**) The motif composition of the *ALDH* gene in *Glycyrrhiza uralensis*. Different colored boxes represent different motifs.

The phylogenetic tree illustrating the expansion of *GuALDH* subfamily members in *Glycyrrhiza uralensis* was created using MEGA. In this phylogenetic tree, most of the subfamily members were grouped closely together, except for the 10 th and 12 th families (Figure 3A). The results show that similar structures have similar functions, which further validates the previous analysis (Figur 2). Genomic DNA sequences were utilized to analyze the gene structure of the *GuALDH* family (Figure 3B). In *GuALDHs*, they contain 4 to 18 exons. The genomic DNA sequences exhibited a length varying from 3500 bp (*GuALDH3F3*) to 11,800 bp (*GuALDH3H2*). Subsequently, the MEME software (Vers. 5.5.1) was employed to investigate the conserved motifs present within the *GuALDH* protein structure. A total of 10 conserved motifs were identified within the *GuALDH* protein. Motif 3 represents a conserved glutamate active site (PS00687) (Figure 3C). The conserved distribution order and the number of motifs indicate that the function of the *GuALDH* subfamily is conserved.

### 2.4. Genome Localisation, Homology, and Evolutionary Analysis of GuALDHs

**Figure 4:**
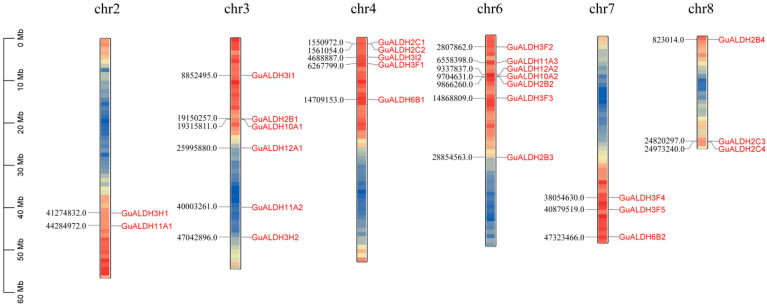
Chromosome distribution of *GuALDHs* in *Glycyrrhiza uralensis*.

To investigate the distribution of *GuALDHs* on the chromosomes of *Glycyrrhiza uralensis*, 26 *ALDH* genes were mapped to six chromosomes. In the genome of *Glycyrrhiza uralensis*, 2 of the 26 *ALDH* genes are located on chromosome 2; 6 on chromosome 3; 5 on chromosome 4; 7 on chromosome 6; 3 on chromosome 7; and 3 on chromosome 8 (Figure 4). The above results suggest that *ALDH* genes on other chromosomes may have expanded based on chromosome 6. Additionally, there are tandemly duplicated gene pairs on some chromosomes, which may lead to the emergence of new biological functions: *GuALDH2B1* and *GuALDH10A1*; *GuALDH2C1* and *GuALDH2C2*. The three tandemly duplicated genes, *GuALDH12A2*, *GuALDH10A2*, and *GuALDH2B2*, are situated on chromosome 6, indicating that this region may have developed new capabilities (Figure 4).

**Figure 5:**
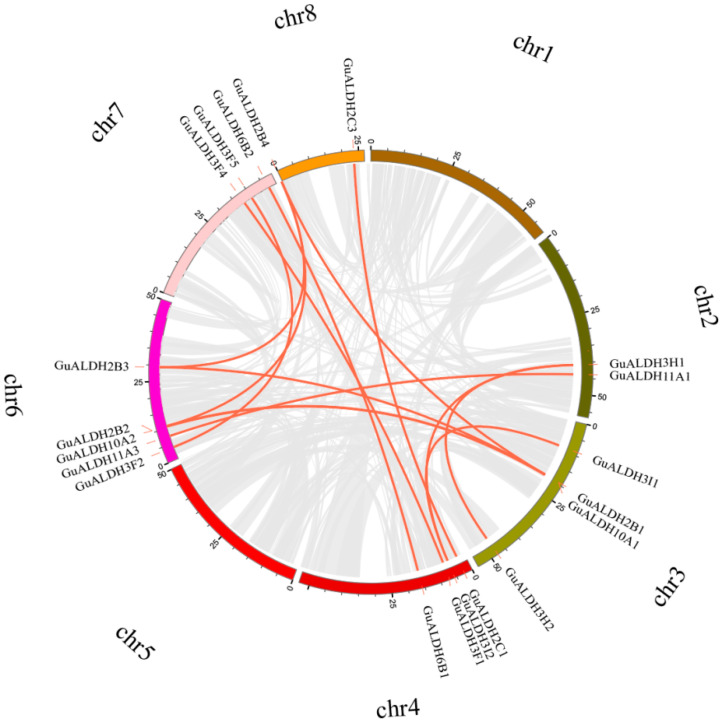
The distribution of the *GuALDH* gene chromosomes and the interchromosomal connections. The connection between duplicated genes in *GuALDHs* is represented by a red line.

**Figure 6:**
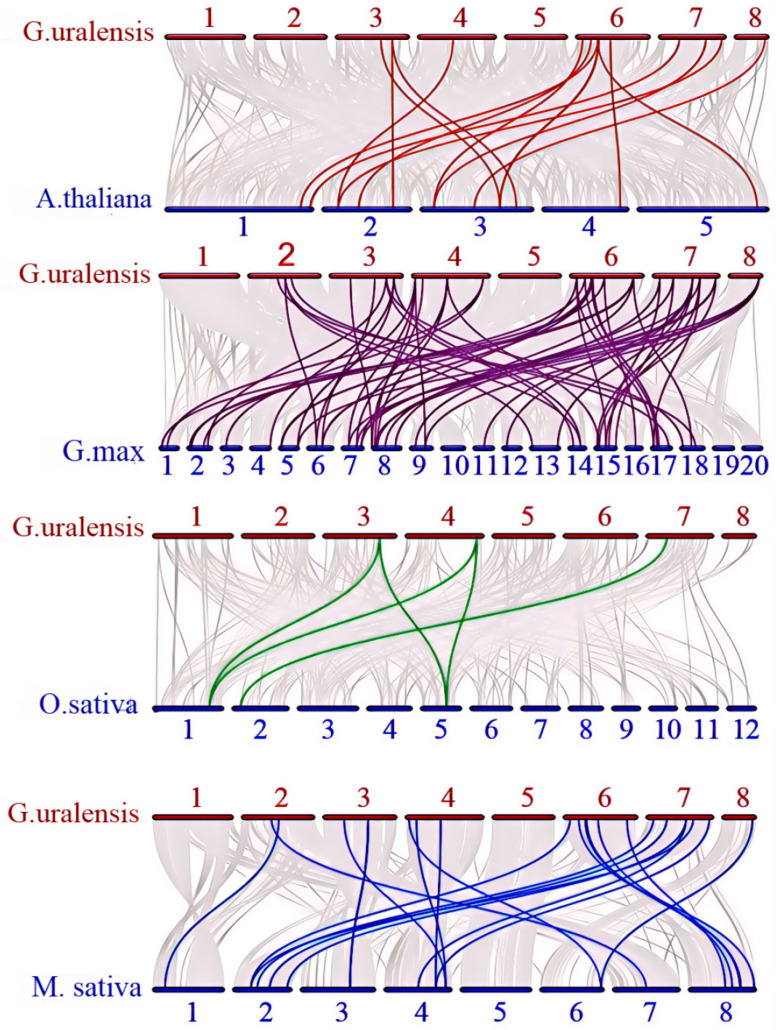
The evolutionary relationship between the *ALDH* gene in *Glycyrrhiza uralensis* and different species of *Arabidopsis thaliana*, soybean, rice, and alfalfa.

As the most important model plants, the genomic functions of rice, *Arabidopsis*, alfalfa, and soybean have been well described. Additionally, soybean, alfalfa, and *Glycyrrhiza uralensis* are affiliated with the legume family. To explore the gene duplication events of *ALDHs* in rice, *Arabidopsis*, soybean, alfalfa, and *Glycyrrhiza uralensis*, we utilized the MCscanX (Vers. 1.0.0) and TBtools software (Vers. 2.056) to establish the homology relationships among these species. The results of the one-to-one comparisons revealed 5 potential homologous gene pairs (Gu-Os) between *Glycyrrhiza uralensis* and rice, 15 potential homologous gene pairs (Gu-Ath) between *Glycyrrhiza uralensis* and *Arabidopsis*, 55 potential homologous gene pairs (Gu-Gm) between *Glycyrrhiza uralensis* and soybean, 20 potential homologous gene pairs in Gu-Ms, and 10 potential paralogous gene pairs (Gu-Gu) between *Glycyrrhiza uralensis* and alfalfa. These findings suggest that these genes have a shared ancestral origin (Figure 5 and Figure 6). Notably, soybean and licorice exhibit the closest genetic relationship, indicating that *ALDH* gene pairs are the most abundant between soybean and *Glycyrrhiza uralensis*.

### 2.5. Identifying Cis-Regulatory Elements in the GuALDHs Promoter

**Figure 7:**
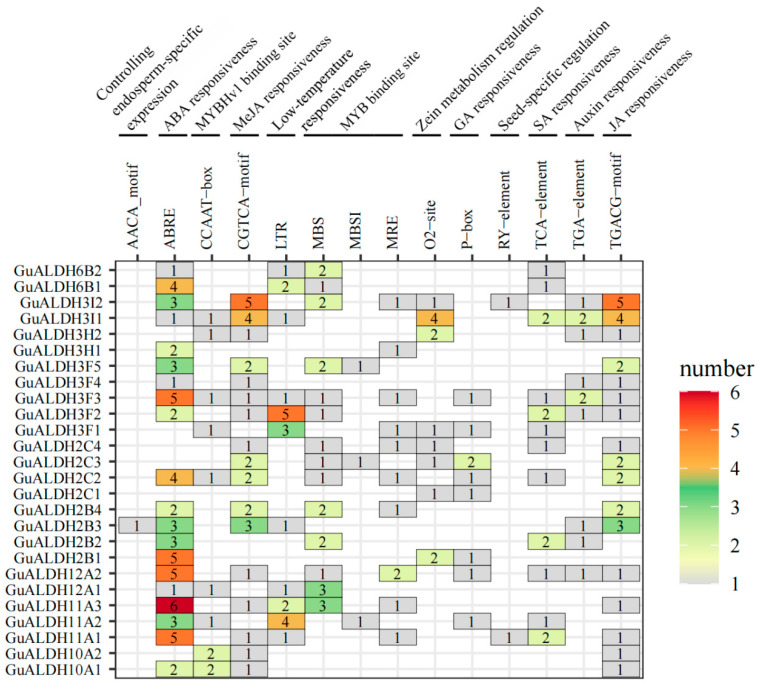
An analysis of cis-acting elements on the promoters of the *GuALDH* gene family in *Glycyrrhiza uralensis*. The sequence 2000 bp upstream of the ATG in *GuALDHs* was analyzed for cis-element responsiveness. The heatmap illustrates the quantity of cis-elements, with higher counts represented in red and lower counts in gray.

In order to investigate the possible biological roles and regulatory mechanisms of *GuALDH* genes, the promoter regions located 2000 bp upstream of 26 *GuALDH* genes were examined to find cis-acting regulatory elements utilizing the PlantCare database. Numerous cis-acting elements were identified, including those associated with the metabolism of phytohormones and biotic stress response, such as drought-responsive elements, salt-responsive elements, abscisic acid-responsive elements, jasmonic acid-responsive elements, salicylic acid-responsive elements, auxin-responsive elements, and other response elements (Figure 7).

The total count of significant cis-elements was recorded, and it was found that there were 61 ABREs (ABA-responsive elements) in the *GuALDH* gene promoter, 30 CGTCA-motifs (jasmonic acid-responsive elements), 9 P-box elements (GA-responsive elements) in the *GuALDH* gene promoter, 11 TGA elements (auxin response elements), and 16 SA-responsive elements within the *GuALDH* gene promoter (Figure 7). Furthermore, most *GuALDH* promoters contained binding sites for *MYB* transcription factors. The aforementioned findings suggest that the *ALDH* gene may have been involved in the regulation of phytohormones and the responses to diverse environmental stresses by modulating various cis-regulatory elements.

### 2.6. Expression Patterns of GuALDH in Different Tissues of Glycyrrhiza uralensis

**Figure 8:**
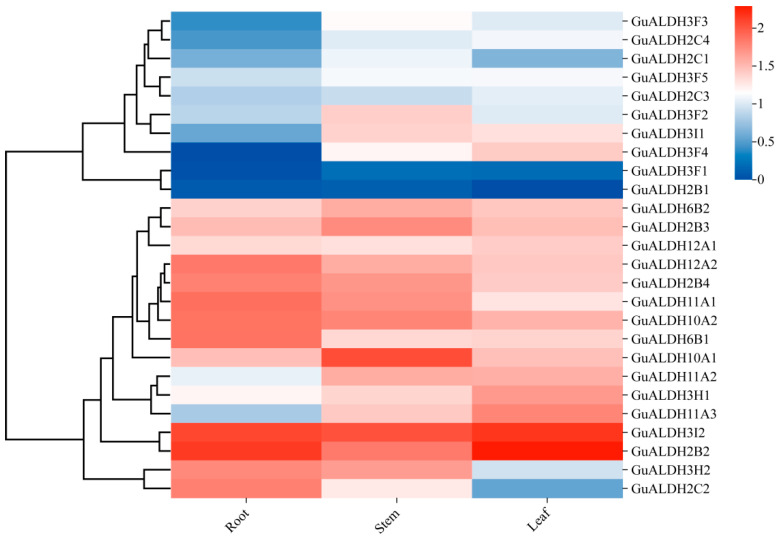
Expression patterns of *GuALDHs* in different tissues. Based on RNA-sequencing (RNA-seq) data of *Glycyrrhiza uralensis*, the expression patterns were analyzed. A hierarchical clustering heatmap was drawn based on the Fragments Per Kilobase of exon model per Million mapped fragments (FPKM) value. The three expression pattern groups are represented by distinct colors: red indicates a high expression level, while blue indicates a low expression level.

The mode of expression of *GuALDH* was analyzed using transcriptome data from various tissue samples of *Glycyrrhiza uralensis* under drought stress (Figure 8). Based on the expression patterns, *GuALDH3F1* and *GuALDH2B1* exhibited negligible or no expression in different tissues. On the contrary, *GuALDH3I2* and *GuALDH2B2* demonstrated high levels of expression in multiple tissues. The above results show that *GuALDH* had different expression patterns, which endowed these genes with different biological functions.

### 2.7. Co-Expression Analysis of GuALDHs

The research findings indicate that soybean is closely related to *Glycyrrhiza uralensis* (Figure 6). Consequently, the co-expressed genes of *ALDH* in *Glycyrrhiza uralensis* were identified by matching the string online database with the soybean genome database.

**Figure 9:**
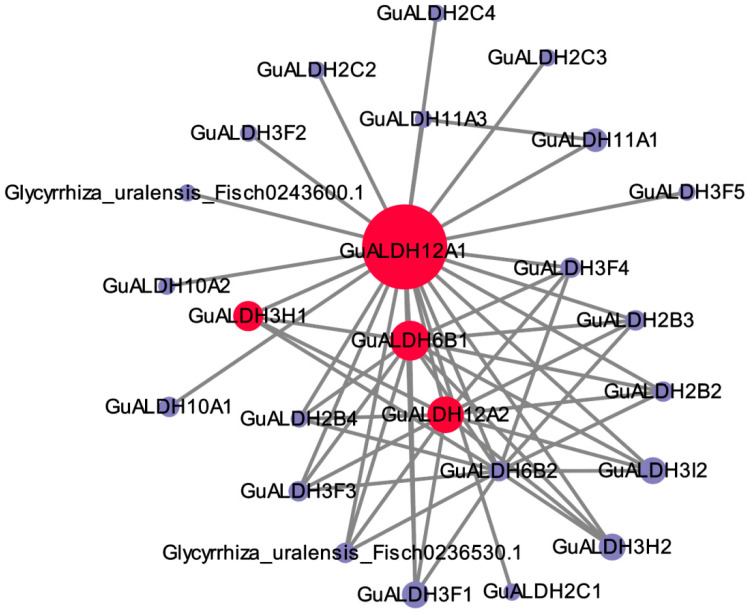
The regulatory network of co-expression for *GuALDH* in *Glycyrrhiza uralensis*.

The *GuALDH* co-expression network consists of 122 nodes and 61 connections (Figure 9). The four genes marked in red, *GuALDH12A1*, *GuALDH3H1*, *GuALDH6B1*, and *GuALDH12A2*, exhibited a high correlation with other genes (Figure 9), and their expression patterns were elevated in various tissues (Figure 8). These results indicate the importance of *GuALDH12A1*, *GuALDH12A2*, *GuALDH6B1*, and *GuALDH3H1* in the response of *Glycyrrhiza uralensis* to drought stress.

### 2.8. Response of GuALDH Genes to Drought Stress

**Figure 10:**
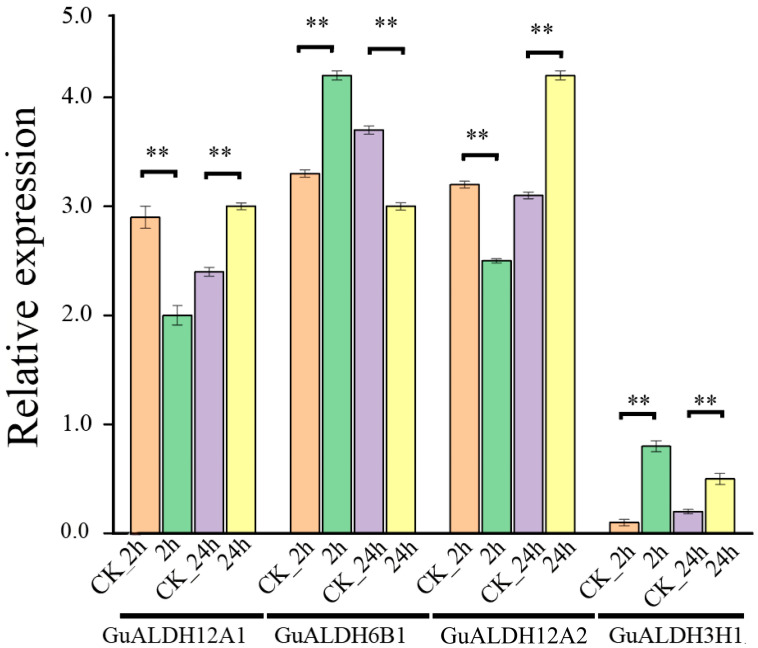
Patterns of expression for *GuALDH* genes in response to drought stress. The relative expression levels of *GuALDH* genes were examined in *Glycyrrhiza uralensis* seedlings after being treated with PEG for durations of 2 h and 24 h. The tap water seedlings were used as a reference for the expression of *GuALDHs* genes at each time point. The data are presented as the mean ± standard deviation. ** *p* < 0.01 indicate statistically significant differences between the two conditions.

To study the role of *ALDH* in environmental stress tolerance, 45-day-old *Glycyrrhiza uralensis* seedlings were treated with PEG (Polyethylene glycol) for 2 h and 24 h. The relative expression levels of the *GuALDH12A1*, *GuALDH12A2*, *GuALDH6B1*, and *GuALDH3H1* genes in *Glycyrrhiza uralensis* were analyzed under drought stress for different times (*p* < 0.01) (Figure 10). At the 24 h mark (*p* < 0.01), the gene expressions of *GuALDH12A1* and *GuALDH12A2* showed a substantial increase in response to drought stress when compared to the control samples, while the results were the opposite after 2 h of drought stress. After 2 h of treatment with drought stress, the expression levels of the *GuALDH3H1* gene were notably elevated in the treatment group when compared to the control sample (*p* < 0.01) (Figure 10). The expression levels of the *GuALDH12A1* and *GuALDH12A2* genes under drought stress for 24 h were significantly higher than those of the control samples (*p* < 0.01), while the results were the opposite after 2 h of drought stress. The expression level of the *GuALDH6B1* gene was significantly higher than that of the control samples after 2 h of drought stress (*p* < 0.01), while the results were the opposite after 24 h of drought stress. Overall, these results indicate that *GuALDH12A1*, *GuALDH12A2*, *GuALDH6B1*, and *GuALDH3H1* respond positively to drought stress by increasing *ALDH* activity, with *GuALDH3H1* playing a more significant role in the response to drought stress.

### 2.9. Changes in Malondialdehyde Content Under Drought Stress

**Figure 11:**
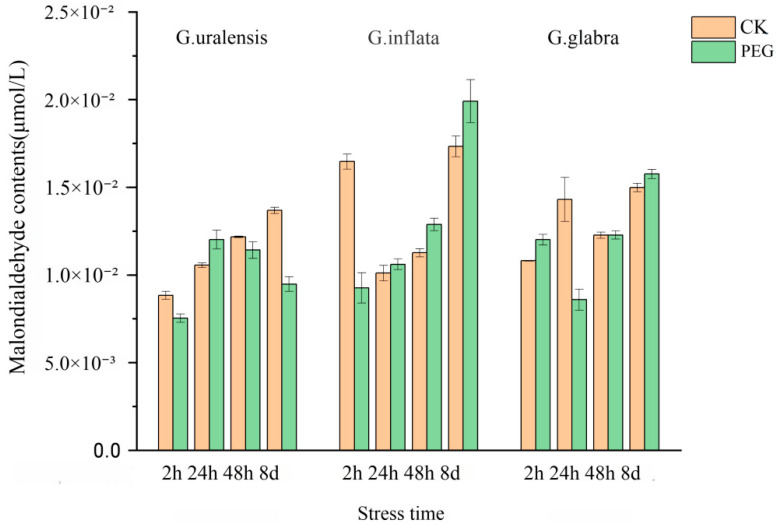
Changes in malondialdehyde content in three kinds of licorice at different times under PEG stress.

The changes in the malondialdehyde content among different species of *Glycyrrhiza uralensis* under the same treatment concentration were analyzed (Figure 11). It was observed that, with an increase in stress duration, there was little difference in the malondialdehyde content between the treatment group and the control group in *G.glabra* and *G. inflata*. However, the malondialdehyde content in *Glycyrrhiza uralensis* under drought stress increased and subsequently decreased. In the treatment group, the malondialdehyde content gradually rose over a short time, but with a prolonged treatment time, it was ultimately found that the malondialdehyde content in the treatment group was lower than that in the control group. The *Aldehyde dehydrogenase* in *Glycyrrhiza uralensis* likely plays a crucial role in eliminating the aldehyde molecules produced in the body under drought stress. In comparison to the control group, this observation indicates that the associated regulatory genes in *Glycyrrhiza uralensis* are up-regulated in response to drought stress, facilitating an increased expression level that helps alleviate the adverse effects of such stress.

## 3. Discussion

Transcription factors play an active role in plants’ responses to abiotic stress, with various mechanisms interacting and collectively promoting plant growth and development. This viewpoint has been confirmed in studies related to different transcription factors, such as *MYB* and *WRKY* [19,30,31,32]. In plants, *Aldehyde dehydrogenase* (*ALDH*) serves as a common “aldehyde scavenger”, which can effectively facilitate the oxidation of diverse aldehydes and aliphatic compounds, thereby promoting their transformation into the corresponding carboxylic acids [33]. The *ALDH* gene family has been studied in a variety of plants, including rice [25], *Arabidopsis* [22], soybean [14], and potato [29]. Investigations concerning the *ALDH* gene family in *Glycyrrhiza uralensis* are limited, although some studies have been conducted on abiotic stress in other leguminous plants.

For example, Wang et al. conducted a study examining the impact of neutral (NS) and alkaline (AS) stresses on the activity of *ALDH* and the expression of *ALDH* genes in common beans. They also analyzed the *PvALDH* gene family using bioinformatics technology [34]. *Glycyrrhiza uralensis*, a traditional Chinese herbal medicine, plays a significant role in the treatment of various diseases [35] and the regulation of immune activity [36]. A total of 53 genes in 10 *ALDH* gene families have been identified in other legumes. In this study, six *ALDH* gene families distributed on six different chromosomes were identified in *Glycyrrhiza uralensis*, comprising a total of 26 *ALDH* genes.

Gene replication constitutes a key link in the process of biological evolution and is the main mechanism for the expansion of gene family and the function evolution of new genes [37], whereas fragment replication and tandem replication are the basis of plant gene family amplification [38]. This study found that tandem duplication and genome-wide duplication (WGD) significantly contribute to the expansion of the *ALDH* gene family in *Glycyrrhiza uralensis*. Two tandem replication events occurred between *GuALDH2B1* and *GuALDH10A1*, *GuALDH2C1*, and *GuALDH2C2*, which may have led to the emergence of new biological functions. In addition, the amplification of *GuALDH* family members is mainly derived from WGD evolution. An analysis of the conserved activation domain suggests that various members of the gene family served distinct roles. In conclusion, the distribution pattern of *GuALDH* in the evolutionary process indicates that environmental conditions and metabolic demands may be key factors in the evolution of *GuALDH* genes.

The expression profiles of genes across different tissues in response to drought stress were examined. It was observed that the expression level of *GuALDH3H1* was significantly higher than that of the control group at both 2 h and 24 h under drought conditions (*p* < 0.01), which aligns with the findings reported for other plant species. In *Arabidopsis thaliana*, the expression of the *AtALDH3H1* gene is up-regulated in response to drought, salinity, and the phytohormone abscisic acid, contributing to its salt tolerance and drought tolerance. In soybean, functional genes related to *GmALDH* are primarily enriched in the proline catabolic pathway [14], highlighting proline’s critical role in responding to abiotic stress. During ABA signaling and dehydration stress response processes, proline regulates ubiquitination pathways or serves as a mediator within metabolic pathways associated with proline [39]. Furthermore, the overexpression of *ScALDH21* in tobacco significantly enhanced reactive oxygen species (ROS) enzyme activity and resulted in the increased accumulation of proline under stress conditions [40].

Under drought stress, ABA acts as a stress signal to regulate gene expression in response to stress. ABER is a cis-acting element of ABA response genes, including the A/GCGT motif [41], which acts as a dehydration-induced promoter in *Arabidopsis*, rice, and soybean to transcriptionally regulate related genes [42]. Studies have shown that nearly half of ABA regulatory genes are affected by drought stress through transcriptome analysis. At present, 245 genes have been identified in *Arabidopsis thaliana* [43], and 43 corresponding genes in rice are regulated by ABA and drought stress [44]. In *Glycyrrhiza uralensis*, *GuALDH11A3*, *GuALDH3H1*, *GuALDH2B1*, *GuALDH12A2*, and *GuALDH11A1* all have more than five ABREs (ABA-responsive elements) and are significantly up-regulated by PEG. The expression of certain genes triggered by ABA was influenced by biotic stress, suggesting that these genes play a role in the stress response through the ABA signaling pathway.

Transcription factors are crucial for plants facing abiotic stress, as various mechanisms interact and work together to promote plant growth and development while responding to external stress. Drought poses a significant barrier to plant growth, involving the interaction between ROS and abscisic acid (ABA) [45]. This study found that the expression levels of *GuALDH11A3*, *GuALDH3H1*, *GuALDH2B1*, *GuALDH12A2*, and *GuALDH11A1* were significantly elevated in the response to drought stress. This suggests that these genes contribute positively to licorice’s ability to withstand abiotic stressors. Different environmental factors restrict the growth and development of plants, and our study establishes a foundational basis for the molecular design and breeding of important traits in *Glycyrrhiza uralensis*.

## 4. Materials and Methods

### 4.1. Genome-Wide Identification of GuALDH Gene in Glycyrrhiza uralensis

To confirm the members of the *ALDH* family within the genome of *Glycyrrhiza uralensis*, our research group utilized cDNA, genome, and protein sequences of *G. uralensis* in conjunction with a Hidden Markov Model (HMM) based on Pfam No. PF00171, which encompasses the conserved *ALDH* domain. The alignment function available in Cluster software (Vers. 3.0) was employed to eliminate sequences that were not present across all eight chromosomes. Additionally, Jalview software (Vers. 2.11.2.4) was utilized to determine whether a conserved glutamine active site (PS00687) is present in *GuALDHs*. The molecular weight (MW) and isoelectric point (pI) of the *GuALDH* protein were determined using ExPaSy (https://web.expasy.org/compute_pi/ (accessed on 8 December 2024)) as a tool, while subcellular localization predictions were made utilizing WoLF PSORT (https://wolfpsort.hgc.jp/ (accessed on 12 December 2024)).

### 4.2. Sequence Alignment and Construction of Phylogenetic Tree

To analyze *ALDH* proteins from *Glycyrrhiza uralensis* in relation to those from the well-researched species *Arabidopsis thaliana*, the protein sequences were aligned with ClustalW. Subsequently, phylogenetic analyses were conducted employing MEGA through bootstrap analysis via the maximum likelihood method and 1000 replicates. A phylogenetic tree was created using Evolview (https://evolgenius.info//evolview-v2/#login (accessed on 19 December 2024)) and subsequently plotted for visualization [30,46].

### 4.3. Gene Structure, Motif Recognition, and Physical Location

In order to analyze the structure of the *ALDH* gene in *Glycyrrhiza uralensis*, TBtools software (Vers. 2.056) was utilized to create exon–intron maps using genomic sequence and coding sequence data. Additionally, conserved motifs were detected with the help of MEME tools (http://meme-suite.org/tools/meme (accessed on 25 December 2024)) in conjunction with TBtools (Vers. 2.056); furthermore, their physical locations on chromosomes were mapped accordingly [31,32,47].

### 4.4. Analysis of Cis-Regulatory Elements of Predicted Promoter Sequences

The genomic sequence of 2000 bp located upstream of the gene promoter ATG was examined, and the PlantCARE database (http://bioinformatics.psb.ugent.be/webtools/plantcare/html/ (accessed on 27 December 2024)) was used to identify potential cis-regulatory elements, including stress-responsive, hormone-responsive, or transcription factor binding elements. We counted and plotted heat maps to show the number of these components.

### 4.5. Homology and Gene Duplication Analysis

To examine the homologous genomic regions, MCScanX (Vers. 1.0.0) in TBtools (Vers. 2.056) was utilized to identify these regions within *Glycyrrhiza uralensis* in comparison to soybean, *Arabidopsis*, and alfalfa. TBtools (Vers. 2.056) was utilized to illustrate the homology of genes. We demonstrated the evolutionary connection of expansion genes by identifying co-linear gene pairs [48].

### 4.6. Gene Co-Expression Networks and Gene Annotation

To analyze the co-expression relationship of *GuALDH* genes, one co-expression gene network of *GuALDH* was identified using the string database, and the co-expression regulatory network was constructed using Cytoscape software (Vers. 3.10.2).

### 4.7. Analysis of ALDH Gene Expression in Glycyrrhiza uralensis Based on RNA-Seq

The expression levels of the *ALDH* gene in various tissues of *G. uralensis* under drought stress was analyzed, and the transcriptome data of *G.uralensis* seedlings treated with 15% polyethylene glycol (PEG6000) were analyzed.

### 4.8. Plant Materials, Drought Stress, RNA Extraction, and qRT-PCR Analysis

*Glycyrrhiza uralensis* is a wild medicinal plant in Tacheng, Xinjiang. The licorice seeds were soaked in concentrated sulfuric acid for 1 h, rinsed with sterile water 7 times, and then sown in a flowerpot containing vermiculite. The temperature was 28 °C/25 °C (day/night), and the illumination period was 16 h/8 h (light/dark). At the same time, *Glycyrrhiza uralensis* roots were gathered and kept for later use at −80 °C. Consistently growing seedlings were chosen, transferred to 300 mL hydroponic bottles, and cultivated for three days in a 1× Hoagland (Na^+^ removed) nutrient solution before being subjected to an abiotic stress treatment [49]. After being treated with 15% PEG6000 for 2 h and 24 h, the samples were frozen in liquid nitrogen and kept for subsequent use at −80 °C in an ultra-low-temperature refrigerator. The total RNA from the seedlings was extracted utilizing the Tiangen RNA plant extraction kit, and the qRT-PCR (quantitative real-time PCR) reverse transcription kit (Toyobo, Tokyo Japan) was used to convert the RNA into cDNA. Gene-specific primers were employed for the purpose of quantitative real-time polymerase chain reaction (PCR). The 2−(∆∆Ct) technique was utilized to evaluate and visualize the produced qRT-PCR data, with the lectin gene found in licorice serving as a reference gene. Table 2 displays the primers that were utilized.

## 5. Conclusions

In the current study, 26 *GuALDH* genes were identified in *Glycyrrhiza uralensis*, which were classified into six families on six chromosomes. Under abiotic stress, the aspects we examined are as follows: gene structure, conserved motifs, cis-acting elements, homologous genomic regions, evolutionary and differentiation patterns, and expression patterns. To summarize, these results offer a thorough theoretical foundation for the *ALDH* gene family in *Glycyrrhiza uralensis* and aid in comprehending the molecular mechanism underlying the *ALDH* gene’s role in the growth, development, and drought stress adaption of this species.

## Figures and Tables

**Table 1:** Basic characteristic of *GuALDHs*.

Family	Gene Name	Gene ID	ORF(aa)	MW(kDa)	pI	Sub Localization
Family2	GuALDH2B1	Glycyrrhiza_uralensis_Fisch0104330.1	492	53.76	6.16	Mitochondrion
	GuALDH2C1	Glycyrrhiza_uralensis_Fisch0121290.1	505	54.63	5.73	Cytoplasm
	GuALDH2C2	Glycyrrhiza_uralensis_Fisch0121300.1	498	54.13	5.99	Cytoplasm
	GuALDH2B2	Glycyrrhiza_uralensis_Fisch0205690.1	542	59.32	8.31	Mitochondrion
	GuALDH2B3	Glycyrrhiza_uralensis_Fisch0227020.1	494	53.51	5.74	Cytoplasm
	GuALDH2B4	Glycyrrhiza_uralensis_Fisch0269090.1	531	57.48	7.99	Mitochondrion
	GuALDH2C3	Glycyrrhiza_uralensis_Fisch0284140.1	501	54.43	6.35	Cytoplasm
	GuALDH2C4	Glycyrrhiza_uralensis_Fisch0284220.1	503	54.96	5.82	Cytoplasm
Family3	GuALDH3H1	Glycyrrhiza_uralensis_Fisch0061860.1	497	54.48	8.83	Cytoplasm
	GuALDH3I1	Glycyrrhiza_uralensis_Fisch0091680.1	545	60.18	7.57	Chloroplast
	GuALDH3H2	Glycyrrhiza_uralensis_Fisch0114530.1	581	64.02	9.39	Peroxisome
	GuALDH3F2	Glycyrrhiza_uralensis_Fisch0197170.1	514	56.60	8.53	Mitochondrion
	GuALDH3F3	Glycyrrhiza_uralensis_Fisch0211780.1	451	50.64	8.45	Cytoplasm
	GuALDH3F4	Glycyrrhiza_uralensis_Fisch0253710.1	470	51.75	7.02	Cytoplasm
	GuALDH3F5	Glycyrrhiza_uralensis_Fisch0257550.1	594	65.54	6.26	Chloroplast
	GuALDH3I2	Glycyrrhiza_uralensis_Fisch0125250.1	545	60.07	6.74	Chloroplast
	GuALDH3F1	Glycyrrhiza_uralensis_Fisch0127260.1	449	50.18	8.88	Nuclear
Family10	GuALDH10A2	Glycyrrhiza_uralensis_Fisch0205450.1	503	54.48	5.18	Peroxisome
	GuALDH10A1	Glycyrrhiza_uralensis_Fisch0104500.1	503	54.78	5.13	Peroxisome
Family6	GuALDH6B1	Glycyrrhiza_uralensis_Fisch0265940.1	510	54.78	6.34	Mitochondrion
	GuALDH6B2	Glycyrrhiza_uralensis_Fisch0138040.1	483	52.19	7.09	Chloroplast
Family11	GuALDH11A1	Glycyrrhiza_uralensis_Fisch0065080.1	519	55.60	7.51	Cytoplasm
	GuALDH11A2	Glycyrrhiza_uralensis_Fisch0113230.1	138	15.39	10.06	Nuclear
	GuALDH11A3	Glycyrrhiza_uralensis_Fisch0201600.1	496	53.14	7.08	Cytoplasm
Family12	GuALDH12A1	Glycyrrhiza_uralensis_Fisch0110500.1	727	79.45	6.87	Cytoplasm
	GuALDH12A2	Glycyrrhiza_uralensis_Fisch0204970.1	544	60.45	6.48	Mitochondrion

**Table 2:** Primers used for *GuALDH* gene expression analysis.

Primer Name	Primer Sequences F (5′-3′)	Primer Sequences R (5′-3′)
GuALDH3F4	TCTCACCGAGCCTAAGGTCA	GACTGAACGCGTCAAACGAG
GuALDH6B1	TTGGCATTGGAAGCTGGTCT	TTGCTGATGACCGGACCAAG
GuALDH12A1	GGTCGCCAAAGGCTCAGATA	CCACCTCTTCCCACCCTAGA
GuALDH12A2	GCCACGGTAGAAGCAGAAGA	TGACCTTGCCAGGAAACGAA
Gulectin	ctgatgcagagcttcaaatcgag	ttcggaaggaaggttgaggtaag

## Data Availability

All data are contained within the article.

## Abbreviations

ALDHs: aldehyde dehydrogenases; ABA, abscisic acid; ROS, reactive oxygen species; HMM, Hidden Markov Model; PEG, polyethylene glycol; RNA-seq, RNA sequencing; qRT-PCR, quantitative real-time PCR; WGD, genome-wide duplication; FPKM, Fragments Per Kilobase of exon model per Million mapped fragments; ORF, open reading frame; aa, amino acid; MW, molecular weight; kDa, kilodalton; pI, isoelectric point.
